# COPD and Comorbid Mental Health: Addressing Anxiety, and Depression, and Their Clinical Management

**DOI:** 10.3390/medicina61081426

**Published:** 2025-08-07

**Authors:** Rayan A. Siraj

**Affiliations:** Department of Respiratory Therapy, College of Applied Medical Sciences, King Faisal University, Al-Ahsa 31982, Saudi Arabia; rsiraj@kfu.edu.sa

**Keywords:** anxiety, biopsychosocial approach, COPD, depression, psychological comorbidities

## Abstract

Anxiety and depression are common comorbidities in patients with chronic obstructive pulmonary disease (COPD), which can contribute to increased morbidity, reduced quality of life, and worse clinical outcomes. Nevertheless, these psychological conditions remain largely overlooked. This narrative review includes studies published between 1983 and 2025 to synthesise the current evidence on the risk factors, clinical impacts, and therapeutic strategies for these comorbidities. While the exact mechanisms leading to their increased prevalence are not fully understood, growing evidence implicates a combination of biological (e.g., systemic inflammation), social (e.g., isolation and stigma), and behavioural (e.g., smoking and inactivity) factors. Despite current guidelines recommending the identification and management of these comorbidities in COPD, they are not currently included in COPD assessments. Undetected and unmanaged anxiety and depression have serious consequences, including poor self-management, non-adherence to medications, increased risk of exacerbation and hospitalisations, and even mortality; thus, there is a need to incorporate screening as part of COPD assessments. There is robust evidence showing that pulmonary rehabilitation, a core non-pharmacological intervention, can improve mood symptoms, enhance functional capacity, and foster psychosocial resilience. Psychological therapies such as cognitive behavioural therapy (CBT), mindfulness-based approaches, and supportive counselling have also demonstrated value in reducing emotional distress and improving coping mechanisms. Pharmacological therapies, particularly selective serotonin reuptake inhibitors (SSRIs) and serotonin–norepinephrine reuptake inhibitors (SNRIs), are commonly prescribed in moderate to severe cases or when non-pharmacological approaches prove inadequate. However, the evidence for their efficacy in COPD populations is mixed, with concerns about adverse respiratory outcomes and high discontinuation rates due to side effects. There are also barriers to optimal care, including underdiagnosis, a lack of screening protocols, limited provider training, stigma, and fragmented multidisciplinary coordination. A multidisciplinary, biopsychosocial approach is essential to ensure early identification, integrated care, and improved outcomes for patients with COPD.

## 1. Introduction

Chronic obstructive pulmonary disease (COPD) is a preventable and treatable, but not curable, chronic respiratory disease characterised by persistent respiratory symptoms and airflow limitations resulting from airway and/or alveolar abnormalities caused by prolonged exposure to noxious particles or gases [1,2]. The main causative factor is tobacco smoke exposure; however, air pollution and occupational and genetic factors are also involved in its aetiology [3].

As one of the current leading causes of death worldwide, COPD is projected to become the third leading cause of death by 2030 [4]. Despite advancements in treatment, millions of deaths related to COPD occur each year, and this rate is projected to grow over time due to ageing populations and increasing exposure to environmental risk factors [4]. The disease places a heavy burden on resources [5], as frequent exacerbations result in increased hospitalisation [6], diminished functional abilities [7], and rising economic costs.

Besides its direct influence on lung function, COPD is becoming increasingly recognised as a systemic disease, with a high frequency of comorbidities, including cardiovascular disease, diabetes, osteoporosis, metabolic syndrome, lung cancer, and psychological disorders, which influence the course of disease and the patients’ quality of life [8]. Although some comorbidities are the result of common risk factors (e.g., smoking or age), others arise from chronic systemic inflammation, oxidative stress, and disruption to the regulation of the nervous and hormonal systems. It is important to mention that a previous study found that more than 90% of patients with COPD have at least one comorbidity [6], with a large proportion of these patients dying from non-respiratory rather than respiratory causes [7].

Anxiety and depression are two of the most common, although regularly underdiagnosed, comorbidities of COPD [2]. These psychological disorders do not directly affect lung function but can have a profound impact on disease progression, symptom perception, treatment adherence, and overall patient outcomes [2]. The relationship between chronic breathlessness and physical limitation and emotional distress forms a vicious cycle that exacerbates both COPD and mental health issues [9].

A growing body of evidence has highlighted the striking prevalence and burden of anxiety and depression across all stages of COPD. The prevalence estimates for anxiety range from 13% to 46% among outpatients and reach up to 55% in inpatients [10]. Their incidence increases with the severity of the disease. For instance, anxiety has been reported in up to 75% of patients with end-stage COPD [11], while depression affects 27% to 40% of stable COPD patients [12], reaching as high as 86% during exacerbations [13]. A previous longitudinal study suggested that COPD patients have nearly double the risk of developing depression compared with matched non-COPD controls, with incidence rates of 11.4 vs. 5.7 per 1000 person-years [14]. Panic disorder and generalised anxiety disorder are also disproportionately represented, with COPD patients being up to ten times more likely to develop panic disorders. Notably, coexisting depression and anxiety affect between 26% and 43% of COPD patients [15,16] and are associated with more severe breathlessness, higher disability, and an increased risk of suicidal ideation. These findings underscore the clinical urgency of identifying and managing these comorbidities throughout the COPD trajectory.

As such, a thorough understanding of mental health disorders is of vital importance in shaping COPD management and the overall well-being of patients. This review synthesises the evidence on the risk factors, clinical implications, management strategies, and practical challenges related to anxiety and depression in COPD. To ensure both conceptual depth and contemporary relevance, this review integrated literature spanning from 1983 to 2025, encompassing seminal studies that have shaped the field alongside the most recent clinical and epidemiological research. This comprehensive approach underscores the necessity of a multidisciplinary framework that addresses both pulmonary and psychological aspects of COPD care.

## 2. Methods and Materials

### 2.1. Literature Search and Review Strategy

**Figure 1:**
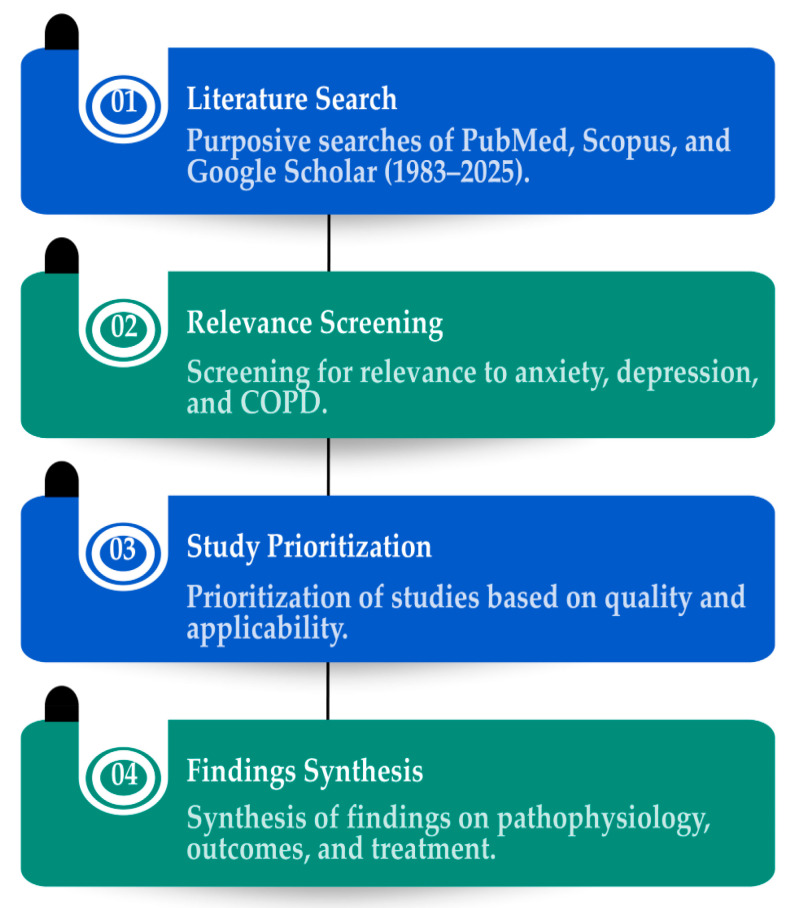
Literature search and review strategy.

This narrative review was designed to synthesise the evidence on the clinical burden, proposed mechanisms linking anxiety and depression to COPD, and management strategies for COPD. To ensure conceptual depth and contemporary relevance, the literature search spanned from 1983 to July 2025, integrating both foundational and current research. A search was conducted using PubMed, Scopus, and Google Scholar. The search terms included combinations of the following terms: “COPD”, “chronic obstructive pulmonary disease”, “depression”, “anxiety”, “psychological comorbidity”, “pulmonary rehabilitation”, “cognitive behavioural therapy (CBT)”, “relaxation therapy”, “antidepressants”, “dyspnea”, “screening tools”, “quality of life”, and “pharmacological treatment”. The selection process is illustrated in Figure 1.

### 2.2. Inclusion and Exclusion Criteria

Studies were eligible if they were peer-reviewed, published in English, and focused on adult COPD patients experiencing anxiety or depression. The types of articles included original research, systematic reviews, meta-analyses, guidelines, and narrative reviews. Exclusion criteria included editorials, commentaries, abstracts without the full text, and studies focusing solely on asthma or non-respiratory conditions.

### 2.3. Data Synthesis

Thematically, data were grouped into five core domains:

- Epidemiological burden of anxiety and depression in COPD;
- Shared pathophysiological mechanisms and bidirectional symptomatology;
- Impact of psychiatric comorbidity on clinical outcomes;
- Non-pharmacological strategies (CBT, PR, and relaxation therapy);
- Pharmacological management and practical limitations.

The evidence was assessed narratively, with an emphasis on real-world applicability, treatment safety, and gaps in current guideline integration. This allowed for a pragmatic discussion of how psychological care might be more effectively integrated into standard COPD management pathways.

## 3. The Intersection of Mental Health and COPD

### 3.1. Understanding Anxiety and Depression

Although anxiety and depression have their own distinct features and clinical presentation, they often co-exist and are common in patients with chronic diseases, such as those with COPD [2]. Although both conditions can severely alter a patient’s feelings, thoughts, and other disease-related aspects, they can invoke different behaviours, perceptions, and physiological effects.

### 3.2. Anxiety: A State of Persistent Worry and Hyperarousal

Anxiety is characterised by excessive fear, heightened alertness, and physical symptoms of autonomic activation [17]. It is often associated with anticipatory worry about future events, which leads to restlessness, muscle tension, difficulty concentrating, sleep disturbances, and avoidance behaviours [17].

Anxiety disorders include many different conditions, such as generalised anxiety disorder (GAD), panic disorder, and various phobias, which can prevent individuals from performing normal activities of daily life [18]. In COPD, anxiety commonly stems from experiencing dyspnoea, fear of exacerbations, and a perceived lack of control over symptoms. It is common for patients to become sedentary or withdraw socially for fear of provoking breathlessness, but these responses can result in progressive deconditioning and loss of function.

### 3.3. Depression: A State of Emotional and Physical Exhaustion

Depression is a prolonged feeling of sadness or emotional or motivational withdrawal [19,20]. While anxiety is associated with high emotional arousal and a heightened state of vigilance, depression is characterised by low energy, disengagement from activities, and cognitive deceleration.

People suffering from depression have, among other symptoms, an overwhelming sense of worthlessness or hopelessness, loss of appetite, disturbed sleep, and difficulty concentrating. In COPD, depression is particularly concerning as it can lead to reduced treatment adherence and pulmonary rehabilitation [21,22], poor self-care [23], and worsening of disease outcomes [2]. The psychological distress of living with a chronic condition and frequent hospitalisations, along with functional limitations, can all directly contribute to depression.

### 3.4. The Overlapping Nature of Anxiety and Depression

Anxiety and depression, despite being distinct conditions, often co-occur in patients with COPD [15,16]. This overlap is clinically significant, as it may impact diagnosis and treatment. Given that patients with both anxiety and depression exhibit worse symptom perception, they are less capable of coping with their disease and have an increased risk of hospitalisation.

The symptoms of anxiety and depression have considerable overlap with those of COPD itself, complicating accurate differentiation in clinical practice. For instance, fatigue, sleeping disorders, and decreased physical activity are features that are shared by both depression and moderate to severe COPD. Moreover, the typical symptoms of panic disorder, such as dyspnoea, palpitations, and a feeling of impending doom, can often mimic exacerbations of COPD.

The specific differences in the main characteristics of anxiety and depression and their clinical importance are enumerated in Table 1, which highlights their relevance in the context of COPD.

## 4. Proposed Mechanisms Linking Anxiety and Depression to COPD

The relationship among anxiety, depression, and COPD is increasingly believed to be both bidirectional and multifactorial, with each disorder worsening the severity and development of the others [9]. This relationship can be explained by a series of biological, psychological, behavioural, and social inputs that together determine the mental and respiratory health of patients suffering from COPD [2].

One key mechanism appears to be chronic dyspnoea, seen as the hallmark of COPD, which drives the reduced exercise capacity and physical deconditioning observed in COPD. Dyspnoea is not only a troublesome symptom but also a strong inducer of both anxiety and depressive symptoms. In a study of 60 stable COPD patients, breathlessness and limited physical function were the primary contributors to emotional distress. These conclusions were further confirmed by a longitudinal study where moderate to severe dyspnoea was found to be a significant predictor for the onset of depression over time [24]. In turn, depression was found to exacerbate the perception of dyspnoea, which suggests a downward spiralling relationship between mental health and respiratory symptomology [25].

Psychosocial factors mediate the relationship between COPD and psychological disorders. The loss of independence, social isolation, and helplessness that patients suffering from COPD usually experience are also significant contributors to anxiety and depression [26]. One Canadian study reported a fourfold higher likelihood of experiencing GAD in those with COPD compared with those without [27]. The association, although somewhat reduced, persisted after controlling for known risk factors and important predictors, including having no confidant, parental domestic violence, and lifetime depressive disorders [27]. Additionally, loneliness—experienced by nearly one-third of COPD patients—has been linked to increased hospital readmissions, poor rehabilitation outcomes, and higher depression rates [28]. The consequences of loneliness, however, are not yet fully understood. These results suggest that early screening and psychosocial support should be integrated into the management of COPD in the hospital setting, utilising a multidisciplinary team. These results stress the importance of early screening and solid psychosocial support, as well as the necessity of a multidisciplinary approach and the inclusion of mental health care in the management of COPD.

Low-grade systemic inflammation may also link these disorders, as proinflammatory cytokines such as interleukin-6 and C-reactive protein have been associated with both COPD and depression. In a large sample of elderly subjects, these inflammatory markers mediated the association of depressive symptoms with pulmonary dysfunction [29]. Chronic inflammation in COPD not only underlies lung pathology but may also influence the neurobiological circuits that modulate mood.

Another bridge mechanism is smoking, a common risk factor for both COPD and depression. Depression is associated with smoking initiation, as well as lower smoking cessation rates, resulting in a longer duration of nicotine use, which further accelerates the development of COPD [30]. Moreover, a lack of physical activity (triggered by depressive symptoms or fatigue related to COPD) exacerbates this downward spiral of deteriorating health and mood [31].

Other social and demographic factors also modify the risk. Women, smokers, and those with a low socioeconomic status have been repeatedly associated with higher rates of depression and anxiety [32,33,34]. Comorbid diseases such as hypertension, arthritis, and cancer, as well as a high rate of hospitalisations, also increase this burden [35]. Pain, although often overlooked in COPD, has been directly associated with both anxiety and depressive symptoms through its adverse effects on sleep, quality of life, and physical activity [29].

**Figure 2:**
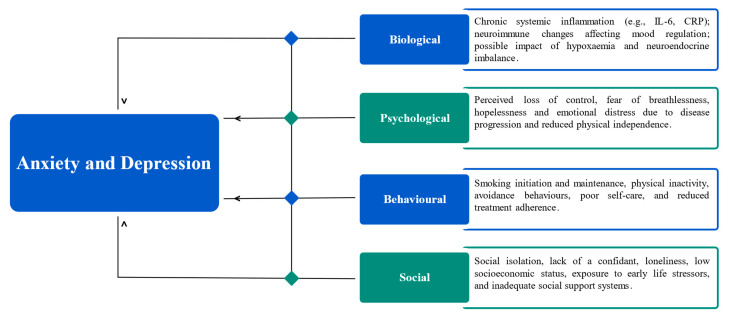
Mechanisms linking anxiety and depression to COPD.

Although these pathways have been well studied, several issues remain. For example, it remains unclear if the observed inflammatory alterations precede or follow the psychological symptoms. Likewise, the effect of hypoxaemia, neuroendocrine dysfunction, and oxidative stress on mood disorders in COPD needs to be studied. The proposed biological, psychological, behavioural, and social mechanisms linking COPD and mood disorders are summarised in Figure 2.

Overall, these results indicate that COPD and psychological disorders are not only comorbid but also have a complex association. Thus, proper management requires a holistic and multidisciplinary approach that addresses the physical, psychological, and social aspects of care.

## 5. Clinical Consequences of Anxiety and Depression in COPD

**Figure 3:**
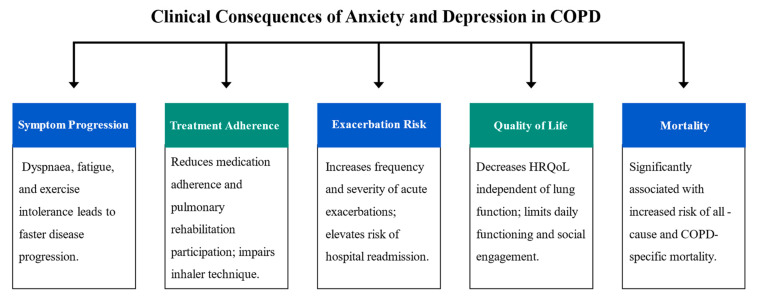
Clinical impacts of anxiety and depression in COPD.

Anxiety and depression heavily influence COPD, leading to the deterioration of respiratory symptoms, poor control of the disease, frequent hospital admissions, a reduced quality of life, and an increased risk of mortality. Such disorders are not only the consequences of COPD but they also increase the burden of the disease. Despite the high prevalence, they are underdiagnosed and undertreated, resulting in poor patient and economic outcomes. The principal clinical implications related to anxiety and depression in patients with COPD are shown in Figure 3.

### 5.1. Worsening of COPD Symptoms and Disease Progression

Anxiety and depression aggravate respiratory symptoms in COPD patients. Studies have shown that individuals with COPD who suffer from these mental health conditions often report greater breathlessness, fatigue, and physical decline, even when their lung function remains stable. Depression is linked to low energy levels, decreased motivation, and inactivity, all of which contribute to muscle deconditioning and exercise intolerance, which in turn lead to more rapid disease progression.

In addition, anxiety and depression tend to increase the perception of dyspnoea, making it feel worse than expected based on measured spirometric or plethysmographic lung function. This distress frequently results in avoidance of activity, as individuals with COPD limit their physical activity for fear of becoming breathless, leading to a further decline in physical conditioning and overall health. There is indeed evidence to show that moderate to severe COPD patients with depression experience a more rapid progression of their disease compared with patients without depression, highlighting that the psychological burden should be considered in the management of COPD.

### 5.2. Reduced Treatment Adherence and Self-Management

One of the most alarming implications of anxiety and depression in COPD is non-adherence to treatment and failure of self-management [36,37]. Patients with depression demonstrate poorer adherence to medication regimens, improperly use inhalers, and are less likely to comply with pulmonary rehab programs [21,22]. This can be attributed to a lack of motivation, cognitive impairment, and feelings of helplessness. Indeed, Volpato et al. (2021) conducted a systematic review on the association between depression and adherence to treatment in COPD patients and found that depressive symptoms were significantly linked to low compliance with medication and poor rehabilitation attendance [38].

This is compounded by anxiety, which leads to avoidance, including missing appointments, undertreatment with prescribed medication through an overconcern about side effects or breathlessness, and, for some, the excessive use of rescue medication. Christiansen et al. conducted a meta-analysis and found that anxiety related to COPD frequently results in maladaptive avoidance behaviour, such as reduced participation in treatment and delayed help-seeking [39]. In another study, highly disease-specific anxiety subtypes, including anticipatory anxiety, were significantly associated with low adherence to inhaled medications in COPD, suggesting an impact of the anxiety profiles on treatment outcomes [40].

Poor self-care behaviours, including inadequate symptom monitoring, avoidance of physical activity, and delayed medical intervention, substantially increase the likelihood of severe exacerbations and COPD-related complications. A previous mixed-methods study showed that a passive or avoidant style of self-care in patients was associated with a greater number of hospital readmissions and declining health status, predominantly due to their delay in seeking medical help and performing daily self-management activities [41]. Pendoni et al. similarly highlighted that physical idleness and the abandonment of daily care are frequent occurrences in the latter stages of the illness and may lead to worsening of symptom control and functioning [42].

Conversely, engaging in active and regular self-care practices is essential to slow the progression of COPD. Evidence has shown that effective self-management interventions, such as symptom monitoring, inhaler adherence, and regular physical activity, are associated with a reduction in exacerbations and hospitalisation rates, as well as improvements in lung function and daily function, with all these positive effects enhancing patients’ quality of life [43,44].

### 5.3. Exacerbations and Hospitalisation

Exacerbations—acute events of deteriorating respiratory symptoms—are a central feature of COPD progression and a leading cause of hospitalisation, accelerated lung function decline, and mortality [45]. Emerging evidence strongly implicates comorbid depression and anxiety as significant contributors to increased exacerbation risk. These psychological comorbidities of patients with COPD also contribute to more severe and more frequent exacerbations.

A prospective, observational study by Martínez-Gestoso et al. showed that depression significantly increased the likelihood of late hospital readmission following acute exacerbation of COPD [46]. This finding has also been reported by Iyer et al., who demonstrated that depression was an independent predictor of 30-day readmissions following exacerbation, which is an indicator of short-term disease instability [47]. In contrast, Coventry et al. demonstrated that readmission risk in patients in the year following an exacerbation-related hospitalisation was related to anxiety and depression in patients who were discharged early [48]. Therefore, identifying and treating these psychological symptoms could significantly contribute to decreasing the risk of exacerbation and promoting clinical stability in COPD patients.

Apart from the short-term clinical consequences, repeated hospitalisations have psychological and economic impacts on both patients with COPD and the healthcare system. The experience of being in hospital, especially during a severe exacerbation, can result in the onset or worsening of anxiety and depression. Patients are frequently subject to painful or arduous procedures, immobility, sleep deprivation, and a fear of death due to respiratory failure, which are factors that also contribute to psychological suffering. This angst may continue beyond discharge in the form of hospital-related stress or depression, and it is part of what has become known as post-hospital syndrome. As highlighted by Ng et al., inpatients with COPD who had depressive symptoms were more symptomatic and had higher readmission rates [49]. Similarly, Laurin et al. found that anxiety symptoms significantly predicted more frequent hospital admissions, suggesting a link between persistent emotional stress and clinical deterioration [50].

The additional psychological distress that develops or is exacerbated during hospitalisation affects recovery and long-term disease control. The decreased motivation and cognitive function associated with depression can also compromise adherence to post-discharge treatment instructions and the ability to recognise early signs of relapse. Anxiety can promote the adoption of avoidant behaviours, such that people will not seek medical care until symptoms become severe. These behaviours lead to an increased likelihood of preventable readmission. Indeed, there is evidence to suggest that anxiety and depression are independent and powerful predictors of early hospital readmission following acute COPD exacerbation, further exemplifying how poor mental health leads to disease instability in a feedback loop [48]. Accordingly, inpatient psychological comorbidity programs and post-discharge interventions with a focus on psychological comorbidities may contribute to diminishing healthcare utilisation and enhancing clinical and psychosocial outcomes in patients with COPD.

### 5.4. Impaired Quality of Life and Functional Decline

Anxiety and depression play a major role in the decline in health-related quality of life (HRQoL) among COPD patients [51]. These extra-psychiatric comorbidities add to the burden of somatic symptoms and lead to a worse physical health status, lower exercise tolerance, and more functional limitation. In a cross-sectional UK primary care study of COPD patients, approximately 21% met the criteria for depression, and 33% met the criteria for anxiety [52]. These comorbidities were significantly associated with higher symptom severity and poorer HRQoL, as evaluated using the EQ-5D visual analogue scale. Most importantly, these relationships were not explained by the severity of spirometry, suggesting that psychological distress impacts are less severe for individuals with more advanced COPD [52].

The systematic review and meta-analysis by Blakemore et al. offers strong longitudinal evidence supporting the association of anxiety and depression with impaired HRQoL in COPD. The review, which included six prospective cohort studies of spirometry-diagnosed COPD patients, demonstrated that depression was significantly associated with HRQoL at the 1-year follow-up based on a pooled estimation from three studies [53]. These results underscore the impact of mental health symptoms on patient well-being, independent of stable pulmonary function.

### 5.5. Increased Mortality Risk

Symptoms of anxiety and depression substantially increase the risk of death in COPD. These psychiatric comorbidities have gained importance as determinants of long-term survival, irrespective of COPD severity and other comorbid diseases.

A landmark population-based cohort study in France conducted by Laforest et al. included 4237 COPD patients aged 45 years and older. Over a 7-year period, 15 comorbidities were identified as predictors of all-cause mortality. Patients with depression comprised 14.2% of patients in the study, and depression was found to increase the risk of mortality by 40% (HR = 1.4 [95% CI: 1.2–1.6]), regardless of the comorbid conditions such as cardiovascular diseases, diabetes, or cancer [54].

A systematic review and meta-analysis by Atlantis et al. also demonstrated that both depression and anxiety increase mortality risk in COPD. This systematic review of longitudinal studies highlighted that the psychological burden in COPD affects patient survival [9]. Similarly, Ng et al. reported that COPD patients who were hospitalised and simultaneously had symptoms of anxiety and depression were nearly four times more likely to die after 12 months [49], thus emphasising the significance of psychological evaluations in clinical settings.

## 6. Screening and Diagnosis: Bridging the Gap in Clinical Practice

**Figure 4:**
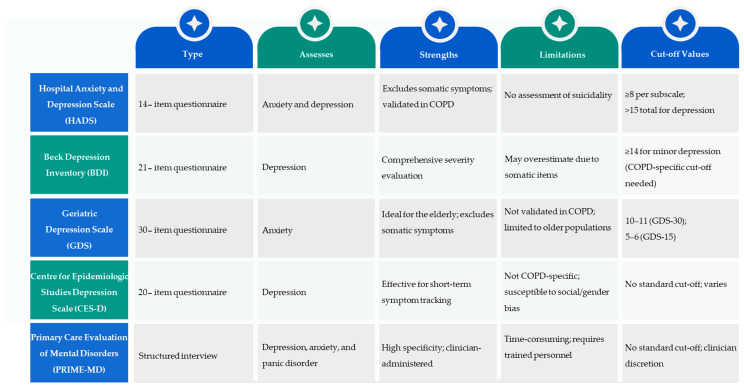
Comparison of commonly used screening tools for anxiety and depression.

Depression and anxiety are common comorbid conditions in COPD that are often underdiagnosed and undertreated in clinical practice. The neglect of these psychiatric disorders may result in marked clinical consequences, such as exaggerated respiratory complaints, higher use of health services, lower adherence to COPD therapy, and increased mortality. Despite this, mental health screening is not a common practice in COPD treatment due to a range of systemic and clinician- and patient-related reasons [55]. Regular and early detection would lead to better patient outcomes, as this would allow treatments to be initiated early and potentially reduce the effects of the chronic psychological burden among COPD patients [56]. Figure 4 shows the most commonly used screening tools for anxiety and depression in COPD based on their clinical focus, validation status, and known limitations.

### 6.1. Available Screening Tools for Anxiety and Depression in COPD

Several validated screening instruments can be employed to measure anxiety and depression in those with COPD. These instruments vary in length, specificity, and applicability to the COPD population. The Hospital Anxiety and Depression Scale (HADS), Beck Depression Inventory (BDI), Geriatric Depression Scale (GDS), Centre for Epidemiologic Studies Depression Scale (CES-D), and Primary Care Evaluation of Mental Disorders (PRIME-MD) are the most commonly used instruments. These instruments have specific benefits and drawbacks that must be taken into account when implementing them in everyday clinical settings for COPD patients.

#### 6.1.1. Hospital Anxiety and Depression Scale (HADS)

The Hospital Anxiety and Depression Scale (HADS) was first introduced by Zigmond and Snaith in 1983 as an interviewer-administered survey for use in nonpsychiatric parts of hospitals to directly measure depression and anxiety. Decades later, it is now commonly utilised in a variety of clinical populations, including those with COPD, in both inpatient and outpatient settings.

The HADS comprises 14 items, which are classified into two subscales: the HADS-Anxiety (HADS-A) and HADS-Depression (HADS-D) scales, each comprising seven items. Notably, the HADS-D excludes somatic symptoms, including fatigue and sleep disturbance, to avoid overlap with physical disease, which is an advantage for chronic illnesses such as COPD. A composite score >15 indicates minor, as well as major, depression symptoms.

There is evidence of the diagnostic and prognostic relevance of the HADS in the management of COPD. Higher HADS scores have been linked to clinically diagnosed depression, with moderate diagnostic accuracy (AUC 0.66–0.68) and validated cut-off thresholds [57]. Anxiety symptoms, which are present in over 50% of hospitalised COPD patients, have also been shown to improve with treatment, underscoring the HADS’s ability to measure responses to clinical treatments [58]. Robust psychometric properties, including high internal consistency and test–retest reliability, further affirm the tool’s stability, particularly for anxiety screening [59]. Additionally, the HADS can identify psychological distress, even in patients with mild COPD, with factors like female gender and BMI emerging as stronger predictors for psychological distress than disease severity. These findings highlight that elevated HADS scores not only aid in identifying underdiagnosed anxiety and depression but can also reflect the symptom burden and monitor treatment responses [60]. Thus, incorporating the HADS into routine COPD care allows for earlier psychological intervention, risk stratification, and a more holistic approach to disease management.

#### 6.1.2. Beck Depression Inventory (BDI)

The Beck Depression Inventory (BDI) was first created in 1961 and has been updated twice, in 1976 and 1996. It is one of the most commonly used instruments for measuring the severity of depression. The revisions have brought the tool closer in line with DSM criteria by substituting more general items (e.g., weight loss and somatic preoccupation) with items reflecting agitation, feelings of worthlessness, and energy loss, which were first implemented in the 1996 revision of the instrument.

The BDI is a 21-item scale (0–70), with 4 items measuring somatic symptoms, including changes in appetite and sleep. It has been extensively validated in patients with post-myocardial infarction, cancer, and diabetes, but it has not been specifically validated in COPD patients (BDI). This restricts its applicability, particularly in the elderly COPD population, whose interpretation of symptoms may differ.

#### 6.1.3. Geriatric Depression Scale (GDS)

The Geriatric Depression Scale (GDS) was first designed as a depression screening tool for older populations and has now found common use in a range of patient and clinical settings, including patients with chronic diseases such as COPD, ischaemic heart disease (IHD), and diabetes mellitus. It can be used in either inpatient or outpatient settings.

The full version of the GDS includes 30 yes/no questions (GDS-30), only one of which is associated with somatic symptoms (fatigue). A 15-item short version (GDS-15) is also available and very useful for frail or low-energy patients. More importantly, the GDS is a tool that focuses on assessing depressive symptoms but not symptoms of anxiety. Although it is widely used in COPD, it has not been specifically validated in this population, which may undermine its interpretative validity. The GDS-30 usually applies a cut-off of 10–11 points for minor depression, and the GDS-15 recommends a cut-off of 5–6 points.

#### 6.1.4. Centre for Epidemiologic Studies Depression Scale (CES-D)

In 1977, L. S. Radloff developed the Centre for Epidemiologic Studies Depression Scale (CES-D), a 20-item self-report questionnaire designed to measure the occurrence of depressive symptoms in the general population. The items represent both the somatic and social aspects of depression but are not completely consistent with the DSM. There is no subscale for anxiety on the CES-D.

The CES-D is widely used in clinical and research practice; however, it has not been specifically tested in COPD populations. This test is more focused on somatic aspects in terms of content and phrasing, and there might be concerns of skewed responses from women and people with autistic traits. For instance, “I had crying spells” may generate gender-based score differences. Furthermore, the somatic aspect of the scale may cause the somatic COPD and depressive symptoms to overlap.

#### 6.1.5. Primary Care Evaluation of Mental Disorders (PRIME-MD)

The PRIME-MD is a clinician-led structured interview that was developed to detect major depression, generalised anxiety, and panic disorders. It includes an easy-to-use patient questionnaire, which leads to a structured clinical interview if symptoms are detected.

While the PRIME-MD is a more complete psychiatric evaluation, it is very time-consuming and not feasible for routine COPD visits. It is best used as part of a system in which mental health professionals can follow up after the initial screening.

The PRIME-MD takes approximately 10–15 min to administer and uses DSM-based diagnostic algorithms that have comparable reliability and validity with more extensive structured interviews. Although not originally developed for COPD, it has been widely used in populations with chronic diseases [61,62]. However, the lack of COPD-specific validation and its focus on diagnosis over symptom severity limits its practicality for ongoing monitoring. Its value lies in its diagnostic accuracy when time and trained staff are available, rather than as a routine screening tool in standard COPD care.

In summary, although several validated screening tools are available, they vary significantly in length, specificity, and feasibility for use in COPD care. The HADS is currently the most practical option due to its brevity and validation in COPD populations. However, tools like the BDI and CES-D may overestimate severity due to somatic overlap, and structured interviews, such as the PRIME-MD, require trained personnel. A significant gap remains in the absence of a screening tool that is explicitly tailored to the psychological and physiological nuances of COPD, emphasising the need for future research and standardisation.

### 6.2. Integrating Mental Health Screening into COPD Care

Although the psychological burden of patients with COPD is being increasingly recognised, structured screening for depressive symptoms and anxiety is rarely performed in general practice. In one cross-sectional study, Siraj et al. found that the rates of formal assessment and use of validated screening measures for depression were as low as 41% and less than 30%, respectively [63]. A combination of system-, clinician-, and patient-level barriers contributes to this critical care gap.

Most patients with COPD are subject to regular follow-ups in primary care, where frontline healthcare professionals can recognise comorbid anxiety and depression. In this high-risk population, targeted mental health screening is justified. Any screening tools must be concise, accessible, and easy to implement within routine respiratory care to be practical and effective. The National Institute for Health and Care Excellence (NICE) guideline advises using two validated questions as an initial approach:

1. During the last month, have you often been bothered by feeling down, depressed, or hopeless?
2. During the last month, have you often been bothered by having little interest or pleasure in doing things?

These items can be included in standard COPD evaluations, and if a positive answer is given, it can be followed by additional investigation [64].

However, the field suffers from a lack of disease-specific tools. There are a few depression and anxiety scales that have been developed for use in elderly populations, but none have been validated for patients with COPD. This lack of standardised, setting-specific tools prevents the consistent application of screening and hinders its clinical applicability [65]. This has led to many clinicians using common scales such as the Hospital Anxiety and Depression Scale (HADS), which is convenient to use and can be administered quickly but may not be sensitive or specific enough in this population. High HADS scores can trigger further secondary screening with broader instruments such as the Beck Depression Inventory (BDI) or PRIME-MD.

## 7. Management Strategies for Anxiety and Depression in COPD: Non-Pharmacological and Pharmacological Approaches

Despite the increased prevalence and incidence of anxiety and depression in patients with COPD and their significant impacts on symptom burden, functional status, and survival, less than one-third of affected patients receive mental health treatment. This treatment gap is also exacerbated by the observation that existing guidelines on COPD provide scarcely any guidance on dealing with psychological comorbidities, often suggesting the same treatment for these conditions as a matched population without COPD, despite overwhelming evidence of the inseparability of mental and physical health in this population.

This mismatch underscores the importance of an interdisciplinary and proactive approach to the management of COPD, which should explicitly include mental health care. Anxiety, depression, and suicidal ideation need to be screened and managed at all healthcare levels, from general practitioners through pulmonologists to psychiatrists, nurses, social workers, and physiotherapists. Pulmonary rehabilitation is a typical example of this comprehensive care approach that not only shows better physical benefits but also less anxiety and depression symptoms, as well as an improved cognitive status.

The optimisation of COPD pharmacological treatment, which includes bronchodilators, corticosteroids, roflumilast, and oxygen therapy, may also have a positive impact on psychological symptoms through improvements in pulmonary function and exercise capacity. However, the lack of psychiatric COPD treatment guidelines makes the task quite challenging, and even more so for psychotropic medications. These treatment modalities should be used cautiously given the risk of drug interactions and the complicated polypharmacy that frequently occurs in the elderly COPD population.

The following section explores the non-pharmacological and pharmacological strategies in detail, highlighting their respective benefits and limitations, as well as the importance of a coordinated, patient-centred approach to achieving holistic COPD care. A summary of the therapeutic interventions for anxiety and depression in COPD is presented in Table 2.

### 7.1. Non-Pharmacological Interventions for Anxiety and Depression in COPD

Non-pharmacological interventions are strongly recommended as a first-line treatment for anxiety and depression in COPD; the evidence for their efficacy can be comparable with that of pharmacological therapies [66]. These interventions, such as CBT and pulmonary rehabilitation and relaxation, aim to alleviate psychological symptoms by emphasising changes in behaviour and lifestyle, thus making them particularly suitable for patients with chronic diseases such as COPD.

Such interventions not only assist in reducing anxiety and depression symptoms but also improve self-management, facilitate better treatment adherence, and target the psychosocial difficulties associated with COPD, such as social isolation and activity limitation. Therefore, they must become part of routine care to facilitate a comprehensive management plan that promotes physical and psychosocial well-being.

#### 7.1.1. Cognitive Behavioural Therapy (CBT)

Cognitive behavioural therapy (CBT) is a first-line, evidence-based treatment option for treating anxiety and depression in individuals with COPD. This time-limited approach focuses on maladaptive cognitions and behaviours that are frequently associated with chronic symptoms such as dyspnoea, disability, and social withdrawal [67]. CBT helps people structure their thoughts and learn how to cope better and regulate their emotions. It can be administered as an individual or group-based intervention, often combined with education and exercise, and is typically delivered by trained mental health professionals. Clinical guidelines recommend the use of low-intensity CBT for subthreshold cases and mild-to-moderate cases, and more powerful interventions (potentially in combination with pharmacotherapy) for moderate-to-severe presentations.

Data from randomised controlled trials and meta-analyses have shown that CBT significantly reduces anxiety and depression symptoms in COPD, with long-term effects and enhanced self-perceived quality of life in many instances [68,69]. However, the TANDEM trial—a multicentre, randomised controlled trial—challenged this consensus with its results from a CBT-grounded psychological intervention delivered by trained respiratory healthcare professionals to people with moderate-to-very-severe COPD [70]. There were no significant differences in anxiety or depression scores at 6 or 12 months compared with usual care, nor in enhanced quality of life, social engagement, or healthcare resource use. While this suggests that the delivery method and patient severity level may influence the effectiveness of CBT, it does not negate the broader body of evidence supporting CBT’s role when delivered by mental health specialists within a multidisciplinary care framework. Thus, while CBT remains a key component of non-pharmacological management in COPD, its implementation must be carefully tailored to the patient’s needs, the severity of their illness, and the qualifications of those delivering the intervention.

#### 7.1.2. Group Psychotherapy

Group psychotherapy seems to be a feasible and cost-effective tool for the treatment of psychological comorbidities in COPD, notably in regions with scarce healthcare resources. Efficient, practical, and requiring fewer therapists, group therapy offers unique healing strengths that enable the treatment of multiple patients simultaneously. According to system dynamics principles, in a group context, healing is fostered through the shared expression of emotions, validation from peers, and attachment to members [71]. Patients may also benefit socially by seeking emotional support, developing self-awareness, and learning social skills from others with similar challenges. Therapists are crucial for such sessions, providing structure and boundaries as they utilise the group process to promote psychological development.

An emerging adjunct to group psychotherapy within pulmonary rehabilitation programs is structured psychoeducation. A recent evaluation of 74 psychoeducation sessions (conducted in a group setting) that were undertaken with 214 participants with COPD found that 95% of patients found the sessions helpful in understanding and managing their symptoms [71]. These sessions helped them identify cognitive triggers and implement simple CBT-informed coping strategies and normalised emotional distress. Emergent themes derived from the qualitative study include improved awareness, skill development, social support, and knowledge of the availability of psychological services [71]. Nevertheless, access to formal psychological support, such as the Improving Access to Psychological Therapies (IAPT) service, was less common and often viewed as difficult to access in practice. These results suggest that psychoeducation within pulmonary rehabilitation will increase psychological insight and empowerment in patients; interventions may be required to ensure that mental health referrals are followed up.

#### 7.1.3. Relaxation Therapy

Relaxation therapy encompasses a diverse array of techniques, including deep breathing exercises, progressive muscle relaxation, mindfulness meditation, guided imagery, and hypnosis, all of which aim to control physiological reactions to stress and improve mental health [72]. Given that the chronic dyspnoea and disability associated with COPD may generate significant distress, these strategies attempt to alter the sympathetic nervous system, improve emotional self-regulation, and create the perception of control. Relaxation techniques are often a part of pulmonary rehabilitation programmes or are used in conjunction with treatments such as cognitive behavioural therapy. A previous meta-analysis of 25 randomised controlled trials involving both inpatient and outpatient COPD populations showed that relaxation had a small but statistically significant positive effect on FEV1, anxiety, and depression, with the most potent effect on quality of life [73]. These results indicate that relaxation interventions could have beneficial psychological and physical effects for COPD patients, and with repeated practice, this effectiveness is likely to be even greater.

In addition to conventional relaxation techniques, other approaches, including Tai Chi, yoga [62], and even singing lessons [63], have also been studied as complementary therapies in the treatment of COPD. A previous study investigated a 12-week combined Tai Chi and yoga (TY) program in older male patients with moderate to severe COPD. The TY intervention produced marked enhancements in pulmonary function, functional fitness, fatigue, and global QOL, with some fitness improvements observed as early as week four [74]. This comprehensive intervention was clinically safe and feasible, suggesting that it could be a supplemental strategy for modulating the psychophysiological aspects of COPD. Integrating sufficiency relaxation, controlled breathing, physical activity, and mindfulness practice, the TY approach is a promising complex therapy in the COPD field. Although more research is required to determine its long-term benefits and generalisability, these observations support the importance of nontraditional “relaxation” treatments in optimising patient outcomes and individualising the management of COPD.

#### 7.1.4. Pulmonary Rehabilitation (PR)

Pulmonary rehabilitation (PR) has emerged as a cornerstone in the non-pharmacological management of COPD, providing a significant number of proven benefits for both physical and psychological symptoms. Although PR has traditionally focused on reducing dyspnoea, improving exercise capacity, and changing functional status, a growing amount of literature suggests that PR may modulate psychological comorbidities, particularly anxiety and depression, which are common in COPD and confer additional morbidity and mortality risks, contribute to disease progression, and impair health-related quality of life [75,76].

PR consists of a combination of supervised exercise, respiratory training, health education, and psychosocial support within a multidisciplinary approach. This overarching management is not only aimed at overcoming physiological limitations but also at alleviating the emotional pain associated with living with a chronic degenerative condition. Through increasing physical function, decreasing fear-avoidant behaviour, and enhancing connections with others in similar situations, PR functions by restoring confidence and control in patients’ day-to-day lives, factors that play a major role in managing psychological distress.

One of the most compelling studies showing the psychological impact of PR was an extensive observational study conducted in 734 patients with stable COPD [77]. Symptomatically significant comorbid anxiety and depression were found to be present in more than a third of participants at baseline. After an 8-week outpatient PR program, significant decreases in the symptoms of anxiety and depression and improvements in dyspnoea, exercise performance, and quality of life were observed in the participants [77]. These findings indicate that patients with a higher baseline psychological symptom burden could derive the greatest emotional benefit from PR. This finding was also strengthened by a systematic review and meta-analysis of six randomised controlled trials showing that comprehensive PR is significantly more effective than standard care in reducing short-term symptoms of anxiety and depression [78]. Significantly, only the programmes that included materials showed psychological benefits; those that did not were associated with detrimental psychological effects, highlighting the importance of an integrated, rather than fractured, model of care. However, the improvements were not maintained at 12 months, suggesting the necessity for guided maintenance programs and studies of long-term strategies.

Further nuance was provided by a prospective Danish study, which investigated PR’s effects on COPD-specific anxiety [79]. Although there was no quantitative reduction in anxiety symptoms, the qualitative interviews suggested that the patients found the therapeutic tools of planning, problem-solving, acceptance, and facing up to fear helpful. These changes were not only due to the content of the PR intervention but also to the contact with healthcare professionals and peers, highlighting the benefits of group interactions and social modelling in a PR setting [79].

Together, these data illustrate PR’s dual role in promoting physiological and psychological recovery. Nevertheless, the best characterised applications of PR seem to be combined applications with specific psychological or pharmacological treatments, particularly for patients with severe or persistent mood disorders. In addition, given that mental health manifestations in COPD can occur through somatic manifestations, a multidisciplinary approach is required for timely diagnosis and holistic management. In this sense, PR is not merely a “rehabilitation” but a transformative care environment that is able to meet the complex needs of people with COPD.

### 7.2. Pharmacological Interventions for Anxiety and Depression in COPD

Pharmacological treatment remains an important option for managing anxiety and depression in COPD, especially in the presence of moderate-to-severe psychological symptoms or when non-pharmacological treatment alone is insufficient. Although the specific data on COPD are limited, current recommendations for managing psychiatric comorbidities follow similar approaches to those for the general population: selective serotonin reuptake inhibitors (SSRIs) and serotonin–norepinephrine reuptake inhibitors (SNRIs) are used as first-line agents, which have good safety and efficacy profiles in medically ill patients. However, questions linger about how medications are prescribed in real-world practice. For example, one systematic review of the use of antidepressants in UK adults with depression found prescriptions to be common even in mild or subthreshold presentations of depression, despite recommendations from NICE to only use them to treat moderate-to-severe depression. This review, which contained fifteen studies, stated that the frequency of use of antidepressants varied from 30.8% to 95.0%, especially in the case of SSRIs [80]. However, there was limited information about the severity of illness or comorbidities, and uncertainty about the extent to which guidelines were being followed and the extent to which drug therapy was evidence-based in some areas of clinical practice. Other alternatives include bupropion (useful for depression and smoking cessation) and mirtazapine, but their specific mechanisms of action and side effect profiles should be considered carefully.

However, the evidence for the effectiveness of antidepressants in COPD is controversial. Some trials have described clinically relevant reductions in the symptoms of anxiety and depression; however, because of methodological concerns such as small sample sizes, heterogeneity, brief follow-ups, and high drop-out rates, the results are uncertain [81]. Furthermore, the adverse events associated with SSRIs and SNRIs include gastrointestinal disturbances, xerostomia, sedation, and psychomotor agitation—symptoms that may be worsened by respiratory symptoms or inhaled therapies in individuals with COPD.

Recent large population-based observational studies raised potential safety concerns for antidepressant use in COPD patients. In a UK self-controlled case series, antidepressant-treated patients had a 79% increased risk of pneumonia and a 16% increased risk of COPD exacerbation within 90 days after treatment initiation, effects that were no longer significant after discontinuation [82]. In an Ontario cohort, markedly elevated risks of hospital admission, emergency department visits, and respiratory-specific and all-cause mortality in older adults initiating SSRIs/SNRIs were detected [71]. Whether these are exposure-induced causal effects or residual confounding effects still needs to be determined, but they underscore the need for vigilant risk–benefit evaluation [83].

Patients who are CO_2_ retainers are at an increased risk of respiratory depression from respiratory suppressants (e.g., benzodiazepines and opiates), and these agents should be avoided or employed with extreme caution [84]. Antidepressants, such as tricyclics and mirtazapine, also merit comment because of their presumed effects on ventilatory drive. In particular, in COPD patients with long-term polypharmacy, increased medical fragility calls for minimally sedating medications with a short duration of action, and prescriptions need to be regularly screened for adverse interactions.

Adherence to medication treatment is another major challenge, with depression itself being recognised as a predictor of non-adherence. A meta-analysis of more than 18,000 patients with chronic illness found that depressed patients were 76% more likely to be nonadherent compared with those without depression [85]. Barriers such as psychiatric stigma, worry about side effects, and pill burden drive the issue. Hence, psychoeducation, regular follow-ups, and a support system that considers both physical and mental well-being are all important in enhancing adherence.

In addition, since dyspnoea often precipitates or amplifies psychological distress [9], targeting breathlessness directly can yield important emotional benefits. Strategies such as optimised bronchodilator therapy, pulmonary rehabilitation, supplemental oxygen, and, in select cases, the cautious use of opioids can play a valuable role in reducing the physical drivers of anxiety and depression, even though they do not directly treat psychological disorders.

Pharmacological interventions for anxiety and depression in COPD patients should be personalised, considering the benefits of treatment and safety issues. While SSRIs and SNRIs comprise the mainstay of pharmacotherapy, their use necessitates vigilant supervision for adverse respiratory events, drug–drug interactions, and difficulties with adherence. Collaborative, interprofessional care—including pharmacological and non-pharmacological modalities—is essential for optimising global health outcomes in this complex patient population.

In short, although a range of interventions exists, a key challenge lies in determining the best treatment for individual patients and how to implement these strategies in routine COPD care. There is evidence supporting CBT and PR as effective first-line strategies, especially when tailored to patient severity and delivered by trained professionals. Relaxation techniques and group psychotherapy may be particularly beneficial in resource-limited settings or where access to individual therapy is constrained. Pharmacological interventions such as SSRIs and SNRIs remain essential in moderate to severe cases but require careful selection and monitoring due to potential respiratory risks. The integration of these interventions into standard care requires a multidisciplinary model, aligning respiratory, psychiatric, and primary care services to deliver the timely, personalised, and holistic management of COPD and its psychological burden.

## 8. Barriers to the Identification and Management of Anxiety and Depression in COPD: Challenges in Clinical Practice

Although the high prevalence of anxiety and depression in patients with COPD is well known, these comorbidities are often not treated, which is associated with a worse health status, more frequent hospitalisations, and a poorer quality of life. A web of barriers, ranging from patient- and clinician-related barriers to systemic barriers [50], lies at the root of the delay in recognising and treating these psychological disorders in routine clinical practice.

At the patient level, the main barriers are a lack of knowledge about depression and anxiety, confusion about somatic and psychological symptoms, and entrenched stigmas about mental illnesses. Many COPD patients may incorrectly attribute decreased psychological well-being to their respiratory status, particularly when symptoms such as fatigue, dyspnoea, and diminished physical functioning are superimposable. This incorrect assumption prevents them from realising or expressing how they feel. Even when recognised, patients might not feel safe discussing their symptoms, be too embarrassed, or think that these symptoms are insignificant compared with their respiratory health. In a recent qualitative study of clinical interviews of COPD patients and their informal carers, similar barriers to structured treatment for mental health problems were identified, including symptom overlap, high physical care burden, stigmas, and mismatched care models [86]. Facilitators, including psychoeducation, self-management support, and person-centred care professionals, were also identified, demonstrating the importance of multidisciplinary, integrated mental health support in COPD pathways. In addition, a preference for care models (e.g., favouring support in primary care or psychotherapy that may not be locally available) can act as a barrier to engaging in treatment.

On the clinician side, time limitations during clinic visits, a lack of psychiatric training, and a lack of confidence in making a psychiatric diagnosis are some of the obstacles encountered [63]. Most providers focus on the physical management of COPD during short office visits, and mood disorders are often left untreated unless patients specifically complain about them. The absence of a uniform COPD-specific method for diagnosing anxiety and depression further aggravates this problem. While instruments such as the PHQ-2, HADS, and BDI are available, and since such instruments incorporate somatic symptoms and cause diagnostic confusion, patients with chronic diseases are vulnerable to being undiagnosed. A lack of follow-up and underutilisation of screening tools can also contribute to missed opportunities for early intervention and continued surveillance.

At the system level, the obstacles include disrupted care pathways, poor integration of respiratory and mental health services, and insufficient insurance coverage for psychiatric care and integrated care. The incorporation of mental health records into electronic health record systems is not uniform, and the priority of some institutions is productivity, measured as the number of patients seen rather than the quality of care. In rural or underprivileged communities, inadequate access to mental health providers compounds these challenges and confines many COPD patients to having no practical access to psychotherapy or psychiatric assessments.

Clinical uncertainty concerning when and how to screen for psychiatric symptoms in COPD is another reason for reluctance. Early identification is crucial, but there is no consensus on whether universal or risk-based strategies are most effective. Adding to this uncertainty is the paucity of evidence regarding the extent to which routine testing for mental health leads to improved outcomes in this population. Furthermore, when depression and anxiety are diagnosed, evidence supporting the efficacy and safety of antidepressants for patients with COPD, especially among the elderly and those with severe disease, is scarce, which limits treatment decision-making and physicians’ confidence.

There are many barriers to identifying, diagnosing, and treating anxiety and depression in COPD patients in clinical practice. Addressing these barriers will require a focused effort to educate and train healthcare providers, implement care systems, and individualise interventions that address the multifaceted conditions of COPD patients with psychological comorbidities. More specifically, integrating psychological care into pulmonary rehabilitation programs can be achieved by embedding mental health screening tools, such as brief instruments like the PHQ-2 or GAD-2, as part of routine intake assessments. Additionally, incorporating low-intensity psychological interventions, such as psychoeducation, mindfulness sessions, and CBT-informed modules delivered by trained respiratory staff, can offer scalable support. However, practical challenges remain, notably the shortage of mental health professionals in respiratory clinics, the limited training of pulmonary teams in delivering psychological care, and fragmented referral systems. Tackling these issues requires cross-disciplinary collaboration, investments in upskilling non-specialist staff, and institutional commitments to holistic, integrated care pathways.

## 9. Conclusions

In summary, anxiety and depression are common but frequently overlooked comorbidities in patients with COPD. Patients with such comorbidities suffer from more severe morbidity and a lower quality of life and have poor clinical outcomes and an increased mortality risk, which are not sufficiently accounted for in clinical practice. Pulmonary rehabilitation has shown success when combined with psychosocial support, while pharmacologic interventions such as SSRIs and SNRIs may be needed for moderate to severe disease. Nevertheless, safety, compliance, and side effects warrant judicious, individualised treatment.

Despite the contemporary awareness that mental health routine screening is not common practice in COPD, the existing tools have limitations, and the issue of structural barriers to screening has not been dealt with (for example, poor integration between primary and mental health care). Primary care practitioners play an essential role in the early recognition and coordination of care, but they require more formalised support and pathways of communication.

Management requires a holistic, multidisciplinary approach that integrates pharmacological and non-pharmacological treatments. Further research is needed to determine the long-term benefits and best delivery models for the treatment of anxiety and depression in COPD patients. Focusing on issues related to mental health in the care of patients with COPD is essential to improving their quality of life and managing the condition effectively.

To enhance clinical application, it is recommended that routine screening for anxiety and depression be incorporated into standard COPD care using validated instruments like the HADS or PHQ-9. Clinical workflows should integrate mental health referrals and foster collaboration among pulmonology care, primary care, and mental health services. Pulmonary rehabilitation programs should consistently include psychological support components. Finally, national COPD management guidelines should explicitly emphasise the importance of addressing mental health comorbidities, supported by provider training and institutional policy changes to facilitate implementation.

## Figures and Tables

**Table 1:** Features of anxiety and depression in patients with COPD.

Feature	Anxiety	Depression
Primary Emotion	Excessive worry, fear, and nervousness	Persistent sadness, hopelessness, and emotional numbness
Cognitive Symptoms	Racing thoughts, overthinking, and difficulty controlling worry	Negative thoughts, self-criticism, and difficulty concentrating
Physical Symptoms	Increased heart rate, muscle tension, restlessness, and shortness of breath	Fatigue, psychomotor slowing, and loss of energy
Behavioural Patterns	Avoidance of feared situations and excessive reassurance-seeking	Social withdrawal and disengagement from activities
Sleep Disturbances	Difficulty falling asleep, frequent awakenings, and nightmares	Insomnia or excessive sleep (hypersomnia)
Appetite Changes	Typically unchanged or increased (emotional eating)	Decreased appetite (weight loss) or increased appetite (comfort eating)
Response to Stress	Hyperarousal and feeling “on edge” or overwhelmed	Reduced motivation and feeling drained or indifferent to stressors
Impact on Daily Life	Impaired decision-making due to overthinking and fear	Decreased productivity and motivation and loss of interest in activities
Common Symptoms in COPD	Fear of breathlessness, panic attacks triggered by dyspnoea, and avoidance of activity	Hopelessness about disease progression, loss of interest in self-care, and increased mortality risk

**Table 2:** Summary of therapeutic interventions for anxiety and depression in COPD.

Symptom Severity	Recommended Interventions	Clinical Applicability
Severe anxiety, major depression, and suicidal ideation	• Pharmacotherapy (selective serotonin reuptake inhibitors (SSRIs)/serotonin–norepinephrine reuptake inhibitors (SNRIs)) • High-intensity psychotherapy (e.g., Cognitive Behavioural Therapy (CBT)) • Inpatient care, with psychiatric referral if suicidal	• Requires specialist care • Monitor for drug interactions and respiratory suppression
Moderate to severe or persistent symptoms	• Combined therapy (CBT + medication) • Collaborative care • Referral to Pulmonary Rehabilitation (PR)	• Combined approaches • Effective within interdisciplinary care models
Mild to moderate subthreshold symptoms	• Low-intensity CBT • Psychoeducation • Relaxation therapy • PR if distress affects function	• Suitable for early-stage or mild cases • Effective in group and primary care settings
Time-limited minor symptoms	• Psychoeducation • Supportive counselling • Monitoring	• Best for situational distress • Often resolves without formal treatment
